# Synchronous Ectopic Adrenal Rhabdomyosarcoma and Ipsilateral Cystic Nephroma Revealing a Diagnosis of DICER1‐Related Tumor Predisposition: A Case Report

**DOI:** 10.1155/cris/2579487

**Published:** 2026-06-15

**Authors:** Alejandro O. Chara, Vikas S. Gupta, Jiancong Liang, Scott C. Borinstein, Daniel J. Benedetti, Julia Shelton

**Affiliations:** ^1^ Department of Surgery, Vanderbilt University Medical Center, Nashville, Tennessee, USA, vanderbilt.edu; ^2^ Department of Pediatric Surgery, Vanderbilt University Medical Center, Nashville, Tennessee, USA, vanderbilt.edu; ^3^ Department of Pediatrics, Division of Pediatric Pathology, Vanderbilt University Medical Center, Nashville, Tennessee, USA, vanderbilt.edu; ^4^ Department of Pediatrics, Division of Pediatric Hematology/Oncology, Vanderbilt University Medical Center, Nashville, Tennessee, USA, vanderbilt.edu

## Abstract

DICER1‐related tumor predisposition (DRTP) results from loss‐of‐function germline variants that predispose patients to numerous benign and malignant tumors. Cystic nephromas are among the most common neoplasms in patients with DRTP; however, there are no reports of rhabdomyosarcoma from adrenal tissue in children with DRTP. We present a case of a 30‐month‐old child with simultaneous cystic nephroma and rhabdomyosarcoma from ectopic adrenal tissue. He initially presented with chronic abdominal pain and poor weight gain. CT imaging demonstrated a large mass extending from the left kidney as well as an adjacent smaller mass in the retroperitoneum. Routine laboratory tests and tumor markers were unremarkable. He underwent an open left radical nephroureterectomy with excision of the midline retroperitoneal mass and lymph node dissection of the retrocaval and paraaortic basins. Pathologic examination revealed the larger mass to be a cystic nephroma, while the smaller mass was found to be embryonal rhabdomyosarcoma arising from ectopic adrenal tissue. Genetic testing of the rhabdomyosarcoma revealed a pathogenic c.4102dup (p.R1368fs) germline frameshift variant and a second c.5425G > A (p.G1809R) somatic missense variant in *DICER1*, as well as a somatic variant in BCOR. With this report, we advocate for further investigation of DICER1‐associated rhabdomyosarcoma to improve management of this rare presentation.

## 1. Introduction

The DICER1‐related tumor predisposition (DRTP) gene plays a critical regulatory role in the synthesis of microRNAs [1]. A los‐of‐function variant in *DICER1* leads to dysregulated gene expression and DRTP [1]. This hereditary cancer predisposition is inherited in an autosomal dominant manner with variable expressivity and reduced penetrance [2]. Patients with DRTP are at increased risk of developing multiple malignant and benign neoplasms during early childhood [3], including pleuropulmonary blastoma, thyroid carcinoma, multinodular goiter, ovarian tumors, sarcomas, and cystic nephroma [2].

Rhabdomyosarcoma accounts for 2%–3% of pediatric cancers, is the most common soft tissue neoplasm in children, and most commonly involves deep soft tissues or striated muscle [4]. Rhabdomyosarcoma in DRTP usually arises in the uterus or cervix [2]. A 30‐month‐old boy presented with synchronous cystic nephroma and embryonal rhabdomyosarcoma arising from ectopic adrenal tissue, revealing a diagnosis of DRTP. This manuscript was prepared following the CARE guidelines.

## 2. Case Report

A 30‐month‐old male with a history of speech delay and strabismus presented to the emergency department with abdominal distention and poor weight gain for 1 month. A palpable abdominal mass that did not cross the midline was noted on physical exam. Laboratory tests, including a complete blood count and basic metabolic panel were within normal limits. CT scan demonstrated a multiseptated cystic left kidney mass measuring 9.5 x 10.8 x 10.4 cm^3^, suggestive of a cystic nephroma. Additionally, a solid 2.5 x 3.0 x 2.5 cm^3^ retroperitoneal mass was adjacent to the cystic mass, thought to arise from a lymph node or adrenal gland. A splenic cyst was also present (Figure 1). Workup for hormone‐secreting adrenal tumors, including cortisol, testosterone, aldosterone, and ACTH, was normal.

**Figure 1:**
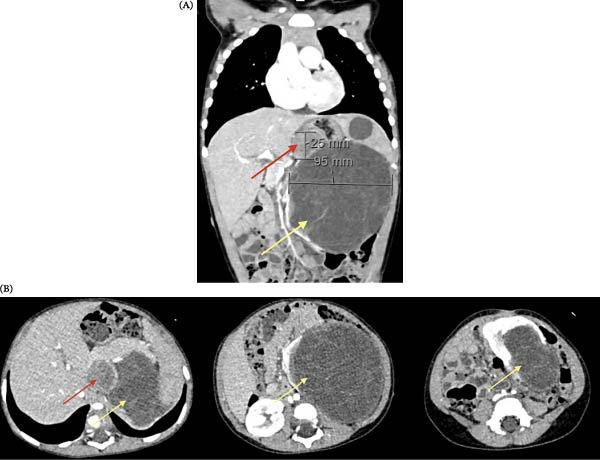
(A) CT chest, abdomen, and pelvis coronal view. The smaller solid mass (red arrow) can be seen medially and superiorly to the large cystic mass (yellow arrow). (B) CT chest, abdomen, and pelvis axial view. The smaller solid mass (red arrow) can be seen medially and superiorly to the large cystic mass (yellow arrow).

He underwent an open left radical nephroureterectomy with excision of the midline retroperitoneal mass and lymph node dissection of the retrocaval and paraaortic basins. The retroperitoneal mass was distinct from the small adrenal gland, without obvious origin. Pathologic examination confirmed the renal lesion as a cystic nephroma measuring 10.5 cm and with negative margins. The smaller, retroperitoneal lesion was an embryonal rhabdomyosarcoma with diffuse anaplasia and heterologous elements. It measured 3.8 cm and had focal lymphovascular invasion with a background of atrophic adrenal parenchyma. The background of adrenal tissue was felt to represent ectopic adrenal tissue since the mass was judged to not arise from the adrenal gland during the operation. This mass did have positive surgical margins for sarcoma. All lymph nodes were negative (Figure 2).

**Figure 2:**
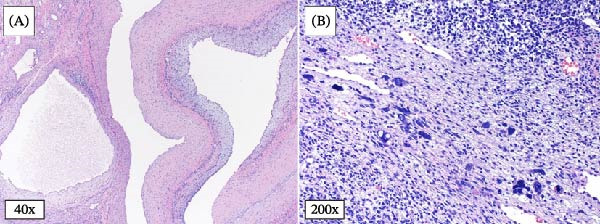
Final operative pathology report. Kidney, left, radical nephrectomy: pediatric‐type cystic nephroma (A). Retroperitoneal lesions, left resection: embryonal rhabdomyosarcoma with diffuse anaplasia and heterologous elements with a background atrophic adrenal parenchyma largely replaced by tumor (B).

Postoperative whole‐body PET scan was negative for metastatic disease. He was therefore staged as group 2, stage 2 fusion‐negative rhabdomyosarcoma, defined as low risk per Children’s Oncology Group risk stratification [5]. He received chemotherapy with 22 weeks of vincristine, dactinomycin, and cyclophosphamide, and adjuvant radiotherapy [6].

By immunohistochemistry, the smaller mass was positive for desmin, myogenin, and MyoD1, and focally positive for Pan‐TRK, while negative for NKX2.2, WT1, PHOX2B, AE1/AE3, S100, SOX10, and calretinin, consistent with rhabdomyosarcoma. The larger mass showed stromal cells in the denser areas that were positive for desmin but were negative for myogenin and MyoD1. This was consistent with cystic nephroma. The rhabdomyosarcoma was negative for FOXO‐1 fusions. Paired tumor‐normal genetic testing confirmed a likely pathogenic c.4102dup (p.R1368fs) (VAF 46.1%) germline frameshift variant in *DICER1*, as well as a second c.5425G > A (p.G1809R) (VAF 45.2%) somatic missense variant in *DICER1*, resulting in biallelic loss of function of *DICER1* in the rhabdomyosarcoma. Somatic testing revealed a BCOR c.1125del (p.Y376fs) (VAF 93.4%) frameshift variant.

Eleven months after his initial operation, the patient had recurrence of rhabdomyosarcoma in the left renal fossa that eroded into the stomach and led to hemorrhagic shock. Despite urgent initiation of chemotherapy and abdominal radiation, he passed away.

## 3. Discussion

DRTP is a rare genetic condition with prevalence ranging between 1:2529 and 1:10,600 that predisposes patients to a large variety of neoplasms [2]. Genetic testing is recommended for those with a tumor known to be associated with DRTP and first‐degree relatives of someone with DRTP [3]. The estimated risk of developing a neoplasm is 5% before the age of 10 and 32% before the age of 60 [3].

Current tumor surveillance guidelines recommend a pelvic ultrasound for gynecologic neoplasms every 6 months beginning at detection of a *DICER1* variant, thyroid ultrasound every 3 years starting at 8 years of age, annual ophthalmologic exams between 3 and 10 years of age, chest radiograph and abdominal ultrasound every 6 months until the age of 8 years, then every 12 months until the age of 12. Chest CT at 3 months and 2.5 years is recommended to screen for pleuropulmonary blastoma. Surveillance of central nervous system, otolaryngological, and gastrointestinal tumors is individualized based on signs and symptoms [3].

Although cystic nephromas occur in up to 10% of patients with DRTP, rhabdomyosarcomas are extremely rare, and no studies have determined their prevalence in this population [2]. Furthermore, DICER1‐associated rhabdomyosarcomas are more often noted to be in the genitourinary system, not arising from the adrenal gland [7].

This case is of a rare embryonal rhabdomyosarcoma arising from ectopic adrenal tissue and cystic nephroma in a toddler leading to the diagnosis of DRTP. There are scattered case reports of adrenal rhabdomyosarcoma, but none in patients with DRTP [8–11]. This report advocates for further investigation into DICER1‐associated rhabdomyosarcoma. We encourage other clinicians caring for patients with DRTP to enroll them in a registry such as the International Pleuropulmonary Blastoma/DRTP Registry to promote our understanding of this rare condition and improve management options.

## Funding

No funding was received for this manuscript.

## Conflicts of Interest

Dr. Scott C. Borinstein serves on a Data Safety Monitoring Committee for Merck. The remaining authors declare that there are no conflicts of interest regarding the publication of this article.

## Data Availability

The data that support the findings of this study are available upon request from the corresponding author. The data are not publicly available due to privacy or ethical restrictions.
